# The Use of Noninvasive Ventilation Outside the Intensive Care Unit: A Clinical Case Report

**Published:** 2013-01-04

**Authors:** A Romano, A Salvati, R Romano, M Mastroberardino

**Affiliations:** 1 Department of Respiratory Medicine, AOSG “San Giuseppe Moscati” Avellino; 2 Department of Imaging, AOSG “San Giuseppe Moscati” Avellino; 3 Department of Medicine, University of Salerno

**Keywords:** Pneumonia, Legionella Pneumophila, Noninvasive Ventilation

## Abstract

Noninvasive Ventilation (NIV) is one of the best weapon at our disposal to treat respiratory failure. The early use of NIV out of the Intensive Care Unit can improve patients’ outcome.

A 58-year-old man affected by severe bilateral pneumonia caused by *Legionella Pneumophila* was treated with Noninvasive Ventilation in extra Intensive Care Unit until the evidence of a marked improvement of clinical and radiological state.

A 58-year-old Caucasian man came to our attention after a five days hospitalization due to fever, cough and progressive dyspnoea previously treated with amoxicillin without any results. In anamnesis, he was a bus driver, no smoker, without relevant history of disease. At the admission at ER, he practiced routine laboratory tests, ECG and chest X-ray that showed the presence of bilateral pulmonary opacities (Figure 1).

After the diagnosis of multifocal pneumonia, he was transferred to the Respiratory Medicine Division. At the clinical observation, the patient was significantly dyspnoeic at rest, presenting cyanosis, cough, profuse sweating in the absence of fever or thoracic pain, confusion, tachypnea but not bradycardia. At the auscultation, there was evidence of bilateral *crepitation* with major involvement of the medium left lung. Without oxygen support the patient showed a SatO_2_ of 62%, that increased to 74% under oxygen therapy (O_2_ 4 lt/min). We performed sampling for Legionella Pneumophila’s urinary antigens and the patient underwent to an urgent chest x-ray. The arterial blood gas analysis showed: pH 7.38, PaO_2_ 33 mmHg, PaCO_2_ 41 mmHg, HCO_3_ 24.3, Sat O_2_ 62%. Blood tests reported: not elevated AST and ALT levels, a mild increased level of creatinine (1.5 mg/dl), a severe hyponatremia (125 mEq/lt), normal leukocytes with an increased level of neutrophils (7.700 /mm^3^; 95%). These results were accompanied by the increased amount of fibrinogen (980 mg/dl), D dimer (3.77 mg/lt), and procalcitonin (8.48 mg/ml) [1].

Two scores were performed to evaluate the patient’s mortality rate: Fine’s score, 147 (V class, mortality rate about 27 %), and Curb-65 score, 3 (one-mount mortality rate 3%).

A high resolution chest CT showed multiple bilateral parenchymal inflammatory foci extended to occupy almost the totality of the left lung and extensive areas of the contralateral lung (Figure 2A).

A treatment was started based on the administration of ceftriaxone, methylprednisolone, clarithromycin, ranitidine, enoxaparin, and mostly Noninvasive Ventilation (NIV).

In Respiratory Medicine Division the diagnosis of Legionella pneumonia was confirmed by the detection of *L. Pneumophila* urinary 1 antigen serogroup and the treatment was confirmed. Since the beginning of this management protocol, the patient showed a gradual improvement, with a progressive reduction of FiO_2_ need.

A follow-up chest CT, performed after 7 days, demonstrated the subtotal regression of the parenchymal foci previously reported, with a residual pleural effusion at the posterior basal left and right middle lobe and unventilated bands at the ventral segment of the upper lobe (Figure 2B).

The Fine’s score decreased rapidly to 57 (II Class, mortality rate 0.9%), and the Curb score was 1 (one-mount mortality about 17%).

After 14 days since the hospitalization, the patient improved and was discharged.

## THE USE OF NIV OUTSIDE THE ICU

Nowadays patients with respiratory failure are frequently ventilated using a noninvasive approach and in many conditions an early treatment with NIV is fundamental to improve the patient’s outcome. The shortage and the high costs of an ICU bed require the use of NIV outside the ICU. The use of NIV should be recommended in ER and in Internal Medicine/ Respiratory Division. Although it is commonly accepted, few patients benefit of a NIV treatment outside the ICU [2,3]. Our aim is to prove the utility of NIV in respiratory failure and the possibility to apply NIV out of ICU without any difficulties even in a severe respiratory failure.

The use of NIV in ordinary wards is necessary for the shortage of ICU beds, the new confidence in NIV management, and the possibility to use NIV in a early phase of the disease [4].

Cabrini et al confirmed the efficacy of NIV outside the ICU, reporting a success rate about 77.5% for NIV outside the ICU in patients with Acute Respiratory Failure (ARF). They showed that the treatment failure was due to complications as nasal decubitus, malfunction of ventilator, incapacity to follow the NIV programme, air leaks from face mask. About 10,1 % of patients were intubated and 12.4 % were dead [5].

Despite these encouraging data, lately Cabrini et al reported that the use of NIV in ordinary wards is reduced by the misperception of the efficacy of NIV among medical team in ordinary wards. Only the 28% of hospitals used NIV in all ordinary wards, even if about the 70 % of them had a NIV programme [4]. Although NIV has been proved to optimize patients’ treatment in ARF, in pulmonary oedema or in post-operative respiratory failure, it is possible to obtain good outcomes where nurse and medical team is trained in NIV protocol [6,7]

The best location for NIV treatment is where there is a staff with training and expertise in NIV, a staff available throughout 24h period, and with the opportunity of a rapid endotracheal intubation and an adequate monitoring. Basic monitoring during NIV requires the clinical observation, the continuous pulse oximetry, the periodical arterial blood gases, and the respiratory rate [6].

The early recognition of those patients that can be advantaged by the use of NIV, and the correct management of the ventilation programme are fundamental to improve the ARF treatment.

## Figures and Tables

**Figure 1.**
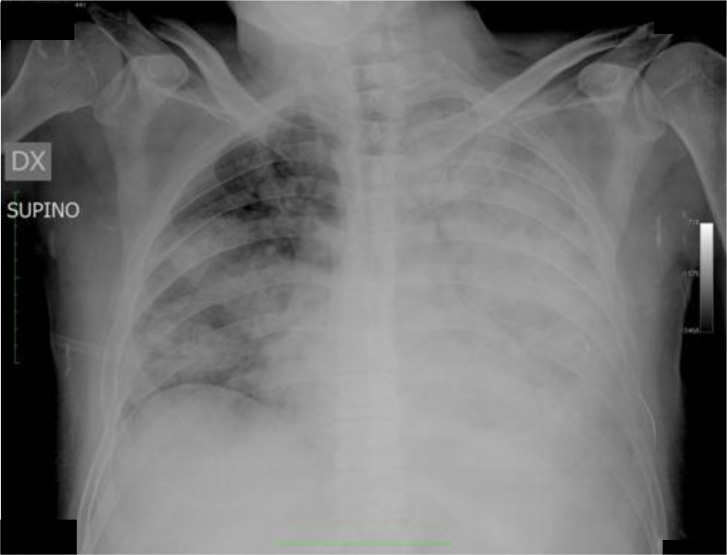
**Chest x-ray at the admission in ER.** Evidence of large bilateral opacities.

**Figure 2.**
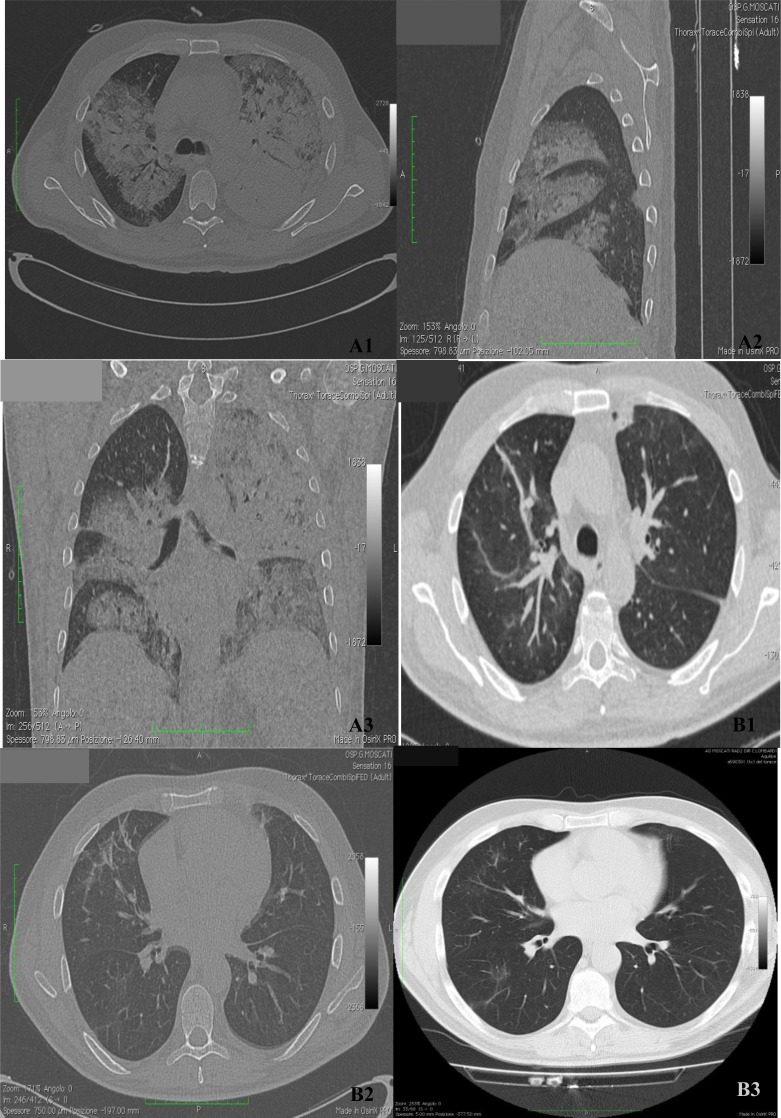
**Chest CT images**. At the ER, the chest Ct highlights the bilateral multiple foci (A). After 7 days there is the important regression of the radiological evidence (B).
